# Gastric cancer in Sub-Saharan Africa – a systematic review of primary data

**DOI:** 10.3332/ecancer.2024.1680

**Published:** 2024-03-07

**Authors:** Anishka Ramadhar, Phoebe N Miller, Mazvita Muchengeti, Juliana Kagura, Kathryn Chu, Cameron Gaskill

**Affiliations:** 1 Division of Epidemiology and Biostatistics, School of Public Health, University of the Witwatersrand, Johannesburg, South Africa; 2 University of California San Francisco, San Francisco, CA, USA; 3 National Cancer Registry, National Health Laboratory Service, Johannesburg, South Africa; 4 Stellenbosch University, Faculty of Medicine and Health Sciences, Cape Town, South Africa; 5 University of California Davis, Davis, CA, USA

**Keywords:** gastric cancer, adenocarcinoma, Sub-Saharan Africa, systematic review, incidence, epidemiology

## Abstract

**Introduction:**

Gastric cancer (GC) is the third leading cause of global cancer-related mortality. Despite the shifting burden of GC to low-and middle-income countries, the data regarding incidence, treatment, and outcomes in these settings are sparse. The primary aim of this systematic review was to aggregate all available data on GC in sub-Saharan Africa (SSA) to describe the variability in incidence across the region.

**Methods:**

Studies reporting population-based primary data on GC in SSA were considered. The inclusion was limited to primary studies published between January 1995 and March 2022 which comprised of adult patients in SSA with GC. Studies without accessible full text in either French or English language were excluded. Unadjusted GC incidence rates with their standard errors for each study were recalculated from the crude numerators and denominators provided in individual studies.

**Results:**

A total of 5,626 articles were identified in the initial search, of which, 69 studies were retained. Reported incidence rates ranged from a high of 5.56 GC cases per 100,000 in Greater Meru Kenya to a low of 0.04 GC cases per 100,000 people in Benin City Nigeria. The overall crude pooled incidence was 1.20 GC cases per 100, 000 (95%CI 1.15–1.26) with a variability of 99.83% (*I*^2^ *p* < 0.001). From the 29 high-quality population-based registry studies the crude pooled incidence was 1.71 GC cases per 100,000 people (95%CI 1.56–21.88) with a variability of 99.60%.

**Conclusion:**

This systemic review demonstrates that GC incidence is highly variable across SSA. The limited data on GC treatment, mortality, and survival presents a significant challenge to providing a complete epidemiologic description of the burden of GC in SSA. There is a need for further robust data collection, exploration, and research studies on cancer care in SSA, with continued assessment of primary data availability.

## Strengths and limitations of this study

### Limitations

- The employed search strategy is open to the biases of the search engines available to authors, potentially missing non-indexed reports published in regional journals.
- Calculations in the study were limited by the availability of regional and population- based estimates that were truly reflective of a given study’s sample population.
- Sparse GC data are available for the SSA countries.
- Histological confirmation was reported in less than 50% of the studies which may have positively skewed the GC incidence. Histological confirmation is needed for accurate data reporting and scarcity of histological information could affect the epidemiology findings for GC incidence.

### Strengths

- This systematic review (SR) included studies which comprised of only primary data.
- This study is the only SR on GC in SSA.
- This study provides the most stringent review of high-quality data available to date to better inform efforts to bolster regional data collection.

## Introduction

Globally gastric cancer (GC) is the fifth most common malignancy with over 1,250,000 new cases diagnosed annually. GC causes over 950,000 deaths each year and ranks as the third leading contributor to global cancer-related mortality [2]. While historically, the burden of GC has been attributed to higher-income countries, the shifting burden of non-communicable disease now attributes over 80% of GC-related deaths to low- and middle-income countries (LMICs) [2, 3]. The World Health Organisation 2020 report on cancer estimated 22,992 incident GC cases within Sub-Saharan Africa (SSA), amounting to over 20,000 deaths per year [4].

Current epidemiologic data on the burden of GC in LMICs is sparse [5]. GC is a multifactorial disease, impacted by genetic and environmental factors that result in wide epidemiologic variation. Up to 20-fold differences in incidence rates have been reported between different geographic regions [8]. Accurate epidemiologic data has historically been dependent on population-based registries. In the absence of high-quality data, cancer incidence, and mortality have been reported by relying on mathematical estimates, which may not appreciate the regional heterogenicity of GC rates or may under-represent GC incidence [9]. This is especially true for SSA, where only 10% of the population is included in population-based cancer registry data collection [10]. Further knowledge of GC incidence, treatment, and outcome is necessary for adequate treatment and public health planning.

The primary aim of this systematic review (SR) is to aggregate all available data on GC in SSA to describe the incidence rates and rate variability of GC in adults in SSA countries. Secondary aims include describing the treatment and mortality of GC in adults from SSA. The hypothesis is that GC is highly variable across the SSA region and this analysis has used primary studies to demonstrate this. The importance of GC variability indicates that the flow of risk factors to disease outcome differs by location. This is critical for public health planning which indicates that treatment and management approaches need to be customised to the variable GC situation at each location. This analysis will allow for a better understanding of the GC disease burden in SSA.

## Methods

### Eligibility criteria

Studies reporting population-based primary data on in SSA were considered. Country inclusion was in accordance with The World Bank definition of SSA. Primary data published between January 1995 and March 2022 which included adult patients in SSA with GC were included. Studies without accessible full text in either French or English language were excluded. All attempts to access texts and data were made, including contacting corresponding authors of publications. There was no involvement from any patients or the public in this SR.

### Information sources, search strategy and study selection

The terms ‘GC’ OR ‘stomach cancer’ OR ‘gastric carcinoma’ OR ‘cancer of the stomach’ OR ‘stomach adenocarcinoma’ were queried when they appeared in the title, abstract or keyword of studies. The names of the SSA countries were applied without language restrictions. A full search strategy is included in Appendix 1. Published studies were identified through a comprehensive search of the following electronic databases; Web of Science, Embase, PubMed and Google Scholar. Duplicate studies were identified and removed. Abstracts were then screened by three authors (AR, PM, CG) to assess eligibility using the predetermined inclusion criteria. Full-text articles were then accessed and reviewed in detail to confirm appropriate inclusion and to extract relevant data. Additional citations were culled and included from the references of articles identified in the initial search. Citations were tracked using Zotero (6.0.8).

### Publication bias and heterogeneity

Risk of bias was limited by maintaining a wide search criterion across multiple high quality electronic databases with a diversity of indexed articles.

### Data extraction

Data collection included the following variables: surname of primary author, publication year, the country where the study was conducted, the study design, diagnostic method, the age group (age range including >18 years old), sample size, the number of GC reported, the overall cancer case number for the study population, the overall population size, regional or national population, differences by age or gender, treatment type, and the case fatality rate of GC in the study. Unadjusted GC incidence rates with their standard errors for each study were recalculated from the crude numerators and denominators provided in individual studies. Where denominators were not available regional and national population sizes were identified using a variety of sources such as macrotends.net, worldbank.org and countryeconomy.com [22–24]. The lowest population size value was used after verifying the values between various sources. Sub-group break downs (age, gender, race/ethnicity) were included. Age standardised rates were recorded without attempts to calculate backwards. Overall mortality and treatment strategies were recorded in crude numbers and percentages. To achieve a high level of reliability, three reviewers (AR, PM, CG) assessed the same articles and reconciled differences before adopting a final and complete data collection document.

### Quality criteria

The strengthening reporting of observational studies in epidemiology (STROBE) checklist was used to assess the quality of the included studies. The final studies included in this SR were assessed using the STROBE checklist and ranked on the following criteria: (1) Inclusion of study in Cancer in Five Continents according to the International Agency for Research on Cancer (IARC) or the data was from a population-based registry [25] (2) 100% histologic confirmation of cancer from a regional registry or national registry (3) >80% histologic confirmation of cancer from a hospital registry (4) histologic confirmation of cancer from any type of registry. The highest rank went to papers included in ‘Cancer in Five Continents’ and regional registries. The second highest rank included regionally or nationally representative studies that were >80% histologically confirmed. The third-ranking category included hospital or pathology registries that showed histological confirmation. The final ranking category included registries that were not histologically confirmed. The quality of studies was given a rank from one to four.

*Data synthesis and analysis*

Unadjusted incidence with their standard errors for each study was recalculated based on the information of crude numerators and either denominators provided by individual studies or the regional population sizes. Regional and national population sizes were identified within the paper and when not reported, they were identified using online sources such as macrotends.net, worldbank.org and countryeconomy.com. Incidence rate patterns of GC across the various nations in SSA after recalculation were summarised. Descriptive analysis was done for the GC treatment and mortality studies. All data was stored in excel (Microsoft Inc, Redmond, WA, USA) analyses were conducted using StataSE (version 15.1, College Station, Texas, USA).

## Results

**Figure S1.**
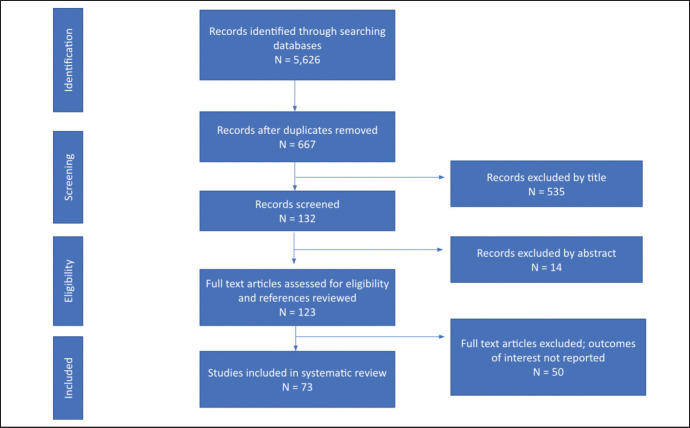
PRISMA flowchart diagram of the study selection.

The database search returned a total of 5,626 articles including primary studies, case reports and reviews. Papers were collected from PubMed (694), Web of Science (4,887), and Google Scholar (45). After duplicates were removed there were 667 unique article titles left. Once the initial screening for titles including incidence of cancer in SSA was complete, 132 studies remained. From the 132 remaining studies, 14 papers were excluded by abstract alone, and the remaining 123 full-text articles were assessed for eligibility. References were searched but did not reveal additional relevant titles. After applying the selection criteria, 69 studies were finally retained in this review (Supplementary Figure S1).

**Figure S2.**
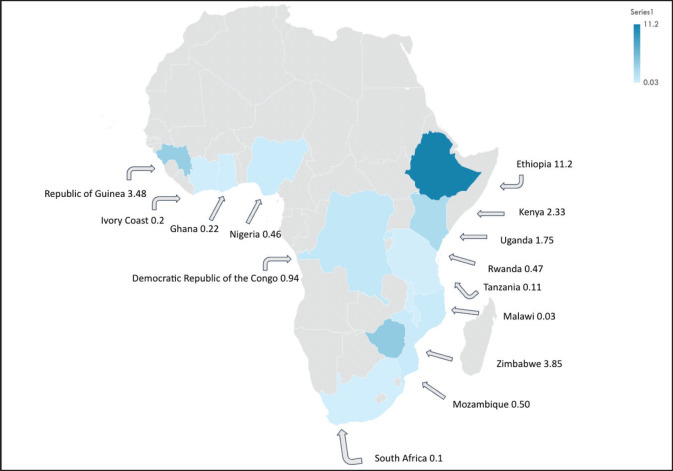
Gastric incidence map with case incidence per 100,000 people^1^.

From the 69 retained studies, we identified that the data collection was conducted across 50 unique study sites (regions or cities) and 23 countries. Most studies were conducted in East Africa [32] with Uganda appearing nine times across six unique locations, Zimbabwe appearing six times in both Harare and Bulawayo and Malawi appearing five times in Blantyre and Lilongwe (Supplementary Figure S2). West Africa had the second most studies [25]; Nigeria produced a total of nine studies across seven separate locations. East Africa and West Africa contributed 43% and 34% of the included studies respectively. North Africa provided studies from Sudan and South Africa provided four studies from Durban and the Eastern Cape (Table 1). Namibia, Botswana, South Sudan, Central African Republic, Chad, Niger and Mauritania are the SSA countries with missing primary GC data (Supplementary Figure S2).

Data was primarily extracted from regional registries, which accounted for 42% (*N* = 31) of the study sites, followed by retrospective reviews of hospital records (40%, *N* = 29). The remaining data were recorded from national registries (9%, *N* = 7) or retrospective reviews of pathology department data (8%, *N* = 6). Of the studies reviewed, 32% (*N* = 24) contained >90% histologically confirmed GC cases. A grading system was implemented to assess the quality of included studies: only 8% (*N* = 6) were from national registries and therefore ranked as the highest quality studies. Most studies (56%, *N* = 42) were from regional or national registries with <80% of the GC cases being histologically confirmed (Table 1).

**Figure 1.**
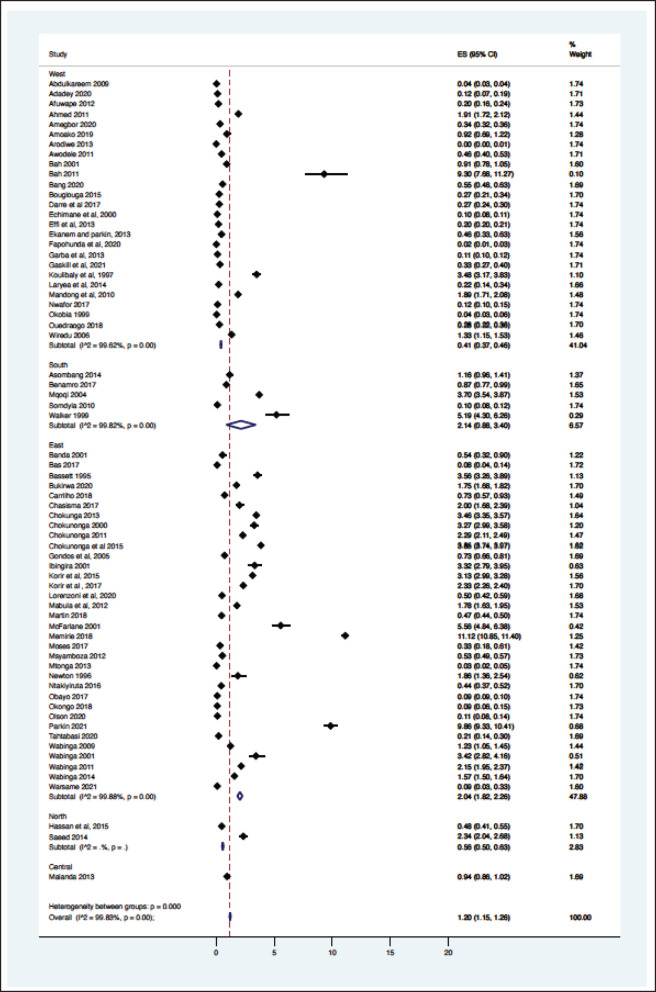
Crude incidence for all data sets describing GC in SSA (*N* = 69).

**Figure 2.**
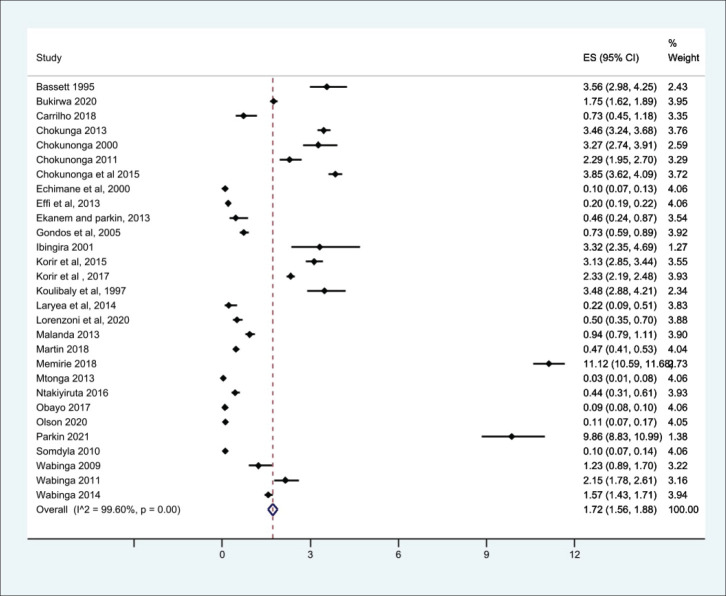
Crude incidence from the high-quality population-based registry studies describing GC in SSA.

From the 69 registry-based primary data sets, the overall crude pooled incidence was 1.20 GC cases per 100,000 people (CI 1.15–1.26). The variability between incidence calculation was 99.83% (*I*^2^ *p* < 0.001) (Figure 1). From the 29 high-quality population-based registry studies the crude pooled incidence was 1.71 GC cases per 100,000 people (95%CI 1.56–21.88) and the variability between studies was 99.60% (Figure 2). The GC incidence variability for West Africa *I*^2^ = 99.62 (*p* = 0.00), Southern Africa *I*^2^ = 99.82 (*p* = 0.00) and East Africa *I*^2^ = 99.88 (*p* = 0.00) indicates significant inter- and intra-regional variability within SSA (Figure 1). The study site with the highest incidence was Greater Meru in Kenya with an incidence of 5.56 GC cases per 100,000 people. The study site with the lowest incidence was 0.04 GC cases per 100,000 people in Benin City of Nigeria (Figure 1).

Twelve (16%) of the studies included treatment information and were primarily from Nigeria (*N* = 3) and Rwanda (*N* = 2). The majority describe either palliation or curative resection (*N* = 11) and a few described adjuncts like chemotherapy (*N* = 4), in Rwanda, South Africa, Tanzania and Cameroon. In Nigeria, between 47% and 86% of patients had surgery with at least half described as palliative.

Twelve (16%) of studies included survival data, with the described time periods ranging from 1991 to 2018 across nine different countries. Median survivals were provided by approximately 33.3% of the studies (*N* = 3), and five studies described absolute survival in 5 years. The median survival periods reported ranged from 4.7 to 13.6 (Table 3).

## Discussion

This SR is the first pooled analysis for GC incidence using primary data from SSA countries. While the pooled analysis shows an average incidence rate of 1.20 GC cases per 100,000 people (CI 1.15–1.26) in SSA, we demonstrate a significant incident variability between individual SSA countries and regions. Most data included in these studies were obtained from hospital registries or hospital data, with <10% included in national cancer registries and <50% containing histological confirmation. Analysis of pooled data limited to high-quality studies reported a similarly low incidence rate (2.12 cases/100,000 people) but retained significant variability. Less than 20% of studies reported treatment or mortality data.

The methodology used in this analysis is similar to other SRs and meta-analyses to estimate cancer incidence in SSA [10, 11], where reliance on population and hospital-based registries leads to similar incidence rate heterogeneity [10, 11]. We found significant heterogenicity (*I*^2^ >99%, *p* < 0.001) among the entire cohort and within geographic regional groups. This finding was maintained when the analysis was limited to only the highest quality data, as identified with the incidence of two East African nations, Ethiopia, and Rwanda with a GC incidence of 11.2 and 0.47 cases per 100,000 people respectively. The GC incidence rates in SSA are comparable to North America and Europe but far lower than Asia. The GLOBOCAN 2020 data shows the GC incidence rates were 5.7 in Northern America, 5.9 in Northern Europe, 4.6 in Western Europe, 8.5 in Western Europe and 22.4 in Eastern Asia where exposure to *Helicobacter pylori* is high and extensive genomic analysis have demonstrated genetic predisposition [31 ,39]. The regional variation of GC is well established, attributed to different risk factors, genetic predisposition, dietary practices, environmental exposures and access to healthcare [3, 4]. *Helicobacter pylori* is a key risk factor for GC and some SSA countries have a known high prevalence of this dependent on multiple factors including geographic elevation, water sources, and sanitation infrastructure [27]. Mozambique and Zimbabwe have a high consumption of smoked, salted, and pickled foods, which are known risk factors for GC [28]. Tobacco consumption is widespread in Ethiopia and Tanzania which may contribute to increased GC among involved populations [29]. Our findings of high incidence variability highlight the importance of local data to better understand the true incidence rate and specific risk factor for any given community.

Namibia, Botswana, South Sudan, Central African Republic, Chad, Niger and Mauritania has missing GC data, the North African and Southern African region provided limited GC data, whilst West and East Africa provided the bulk of the data used in this analysis. Nigeria, Malawi, Zimbabwe, and Uganda provided most of the data. This speaks to the longstanding, high quality successful cancer registries in SSA, including the Harare, Kampala and Eastern Cape population-based registries [13, 16, 17]. The large gap from missing data contributes to the true incidence of GC in SSA remaining unknown. A dire need for these registries to be replicated in other African countries remains, highlighted by the geographic variability in cancer epidemiology and existing gaps in GC diagnosis and care in SSA. The data quality is vastly different among SSA countries, closing the data gap and standardising the quality of data collection will minimise the incidence bias that is currently present for GC in SSA [38].

This SR incident results are similar to rates previously reported by modelling estimates [1]. The 2020 IARC (GLOBOCAN) estimated a SSA GC incidence rate of 2.1 cases per 100,000 people [7, 8]. Likewise, Institute for Health Metrics and Evaluation’s (IHME) Global Burden of Disease estimates a crude incidence rate of 3.21 cases per 100,000 [21]. The overall reported incidence rates are similar to these modelled, validating the accuracy of the average over the studies included. GLOBOCAN and IHME used different models to produce estimates of GC rates as a means to overcome the known limitations in primary data availability and quality on GC in SSA. However, these modelling methodologies are limited by the validity of assumptions regarding disease incidence and may not reflect the true disease burden in the population [26]. The GLOBOCAN methodology derived incidence rates from available registry information or from the average of rates from neighbouring countries, extrapolated over the known population of an entire country [8]. Only 12% of the countries in SSA had national data for these estimates, with 36% of countries incident rates relying on neighbouring country data [9]. The IHME rate was produced by estimating mortality rates for GC through registry information and then dividing by modelled mortality-to-incidence ratios. While this methodology maximizes data coverage, it is based on reported case fatality rates, which may overestimate incidence in areas with worse than expected mortality. The methodology utilised in this SR was stringent as only primary data were included in the analysis and regional and national population sizes were verified with global population databases [22–24]. While our results are comparable to the modelled estimates over SSA as a whole, they have the added benefit of improved GC incidence characterisation within subregions as evident by the high heterogenicity of reported rates [26]. These metrics may provide a more precise understanding of localised disease trends upon which disease prevention and management approaches may be planned.

In most SSA nations, access to healthcare is limited with decreased treatment capacity leading to increased mortality from GC [30]. Less than 20% of all the studies provide information for treatment or survival. From the available data, most surgeries reported were palliative. This is possibly a result of poor surveillance, late-stage presentation, and lack of treatment options upon presentation [5]. Late-stage GC diagnosis, healthcare costs, inadequate diagnostic tools and limited healthcare workers all contribute to restricted GC treatment in SSA [5, 6]. The African enigma which hypothesizes the disassociation between GC and *H. pylori* infection is important to consider in further research [108]. The scarcity of robust data for GC in SSA, and the association between *H. pylori* and GC in SSA not being widely researched may indicate the African enigma is not as convincing as prior belief [108, 109]. Despite these limitations, treatment data are essential to establishing context-relevant treatment guidelines [33]. Future data collection could focus on the current standard of GC care in SSA and available palliative services.

Population-based GC disease registries such as the successful Harare, Eastern Cape and Kampala registries identifies demographic and geographic discrepancies in incidence and mortality rates which is valuable for improving cancer care and developing population specific control measures [34, 37]. Granular data collected from hospitals, clinics and laboratories will give better clarity of the epidemiology and risk factors that shape the local epidemiology of GC. This information on GC in SSA is vital to achieve a better understanding of the local and regional disease landscape and demonstrate the impact of local public health improvements accounting for the high regional variability of GC in SSA [35]. Establishing and maintaining population-based registries at local levels pose a challenge to most SSA regions due to limited healthcare infrastructure, shortage of trained data collectors, high costs, time involvement, and accuracy needed for proper data collection [36]. Disease registry success may be possible by focussing on local-level data collection that is aligned with and monitored by national data collection guidelines to ensure adherence to high-quality standards. Regional and national health systems will benefit from investing in GC surveillance programmes, national control programmes and training of health workers for accurate data collection [34, 40].

This review was limited by several factors. The employed search strategy is open to the biases of the search engines available to authors, potentially missing non-indexed reports published in regional journals. Additionally, our calculations were limited by the availability of regional and population-based estimates that were truly reflective of a given study’s sample population. Despite these limitations, we provide the most stringent review of high-quality data available to date to better inform efforts to bolster regional data collection. Moreover, we likely underestimate the true burden of GC disease in SSA as the studies that do exist are limited by selection bias for those who receive care for GC.

## Conclusion

This study demonstrates the high variability of GC incidence across SSA, independent of data quality. We calculated an overall rate of 1.20 cases per 100,000 people, similar to modelled estimated but highlighting the large regions devoid of primary data. The variable GC incidence rates from the contributing studies of this SR highlight the need for further development of population-based cancer registries [19, 20].

## Conflicts of interest

Our authors have no competing interests and the contents of this manuscript have not been published elsewhere. There are no conflicts of interest to disclose.

## Funding

PROSPERO number CRD42022341498.

No external funding or funding by any author has been received for this study

## Figures and Tables

**Table 1.** Characteristics of included studies describing GC incidence in SSA.

Reference number	Author	Country	Region/City	Study period	Regional population	Study cancer population	GC cases	% Histologically confirmed GC cases	Registry type	Age group	Quality rank
[41]	Abdulkareem *et al* 2009	Nigeria		1995–2006	220,000,000	713	78	100	Regional	All	2
[42]	Adadey *et al* 2020	Ghana	Volta	2012–2014	6,900,000	567	8	0	Regional	All	3
[43]	Amégbor *et al* 2020	Togo		1984–2008	112,000,000	5,251	379	100	Pathology	All	3
[44]	Afuwape *et al* 2012	Nigeria		2004–2009	25,000,000		49	89	Hospital	Adults	3
[45]	Ahmed *et al* 2011	Nigeria	Zaria	1995–2009	9,375,000		179	100	Hospital	Adults	2
[46]	Amoako *et al* 2019	Ghana		2015	2,616,000	736	24	0	Regional	All	3
[48]	Arodiwe *et al* 2013	Nigeria		1995–2010	158,000,000	335	4	0	Hospital	Adults	4
[49]	Asombang *et al* 2014	Zambia	Lusaka	2010–2012	4,384,000		51	100	Hospital	All	3
[50]	Awodele *et al* 2011	Nigeria	Lagos, Oyo	2005–2009	48,000,000	5,094	221	0	Hospital	All	4
[51]	Bah *et al* 2001	Gambia		1988–1997	10,152,000	2,957	92	21	National	Al	3
[52]	Bah *et al* 2011	Gambia		1993–1997	559,000		52	19	National	All	3
[53]	Banda *et al* 2001	Malawi	Blantyre	1994–1998	2,600,000	2,259	14	39	Regional	All	4
[54]	Bang *et al* 2020	Cameroon		2013–2018	19,000,000	1,105	105	100	Hospital	All	3
[55]	Baş *et al* 2017	Somalia		2016–2017	14,190,000	403	11	0	Regional	Adults	4
[57]	Bassett *et al* 1995	Zimbabwe	Harare	1990–1992	3,400,000	2,716	121	50	Regional	All	1
[58]	Benamro *et al* 2017	South Africa	Durban	2009–2014	15,000,000		131	100	Hospital	All	3
[60]	Bouglouga *et al* 2015	Togo	Lome	2005–2012	11,200,000	250	30	100	Hospital	All	3
[13]	Bukirwa *et al* 2021	Uganda,	Kampala	1991–2015	38,007,000	31,357	665	0	Regional	All	1
[61]	Carrilho *et al* 2019	Mozambique	Maputo	2015–2016	2,200,000	1,705	16	76	Regional	All	1
[62]	Chasimpha et al 2017	Malawi	Blantyre	2008–2010	2,046,000	3,711	41	100	Regional	All	2
[63]	Chokunonga *et al* 2013	Zimbabwe	Harare	1991–2010	27,000,000	28,319	933	0	Regional	All	1
[63]	Chokunonga *et al* 2000	Zimbabwe	Harare	1993–1995	3,696,000	3,571	121	59	Regional	All	1
[64]	Chokunonga *et al* 2011	Zimbabwe,	Harare	1993–1997	6,278,000	2,300	144	67,4	Regional	All	1
[65]	Chokunonga *et al* 2015	Zimbabwe	Harare	1991–2010	27,000,000	37,787	1,040	0	Regional	All	1
[65]	Darre *et al* 2017	Togo	Lome	2009–2016	54,968,097	1,738	147	100	Hospital	All	3
[66]	Echimane *et al* 2000	Ivory Coast	Abidjan	1995–1997	38,007,000	2,815	37	0	Regional	All	1
[67]	Effi *et al* 2013	Ivory Coast	Abidjan	1984–2009	384,000,000	12,841	782	100	Regional	All	1
[68]	Ekanem and Parkin 2016	Nigeria	Calabar	2009–2013	1,967,000	719	9	100	Regional	All	1
[70]	Fapohunda *et al* 2020	Nigeria	Lagos	2015	50,800,000	548	9	0	Hospital	All	4
[72]	Garba *et al* 2013	Niger		1992–2009	212,000,000	7,031	236	42	National	All	3
[73]	Gaskill *et al* 2021	Ghana		2014–2015	30,000,000	2,562	98	0	Hospital	Adults	4
[74]	Gondos *et al* 2005	Uganda	Kampala	1993–1997 (2002 follow-up)	12,500,000	1,831	91	48	Regional	Adults	1
[76]	El Hassan *et al* 2015	Sudan	Khartoum	2000–2004	18,450,000	1,958	88	100	Hospital	Adults	3
[77]	Ibingira 2001	Uganda	Kampala	1995	964,000		32	100	Hospital	Adults	1
[80]	Korir *et al* 2015	Kenya	Nairobi	2004–2008	13,922,000	8,982	436	83	Regional	All	1
[81]	Korir *et al* 2017	Kenya	Nairobi	2000–2014	43,700,000		1,019	0	Regional	All	1
[82]	Koulibaly *et al* 1997	Guinea	Conakry	1992–1995	3,043,300	2,064	106	0	National	All	1
[83]	Laryea *et al* 2014	Ghana	Kumasi,	2012	2,300,000	253	5	74	Regional	All	1
[84]	Lorenzoni *et al* 2020	Mozambique	Beira and Maputo	201–4–2017	6,629,000	4,373	33	70	Regional	All	1
[6]	Mabula *et al* 2012	Tanzania	Mabula	2006–2011	13,000,000	5,134	232	100	Hospital	Adults	3
[93]	Nsondé Malanda 2013	Congo	Brazaville	1998–2009	14,428,000		135	0	Regional	All	1
[85]	Mandong *et al* 2010	Nigeria	Plataeu State	1985–2004	10,862,000	5,705	205	100	Hospital	All	3
[85]	Martin *et al* 2018	Rwanda		2012–2016	51,000,000		229	100	Hospital	All	1
[86]	McFarlane *et al* 2001	Kenya	Greater Meru	1991–1993	3,600,000		200	0	Hospital	Adults	4
[87]	Memirie *et al* 2018	Ethiopia	Addis Ababa	2012–2015	14,539,000	64,285	1,617	89	Regional	All	1
[89]	Moses *et al* 2017	Malawi	Lilongwe	2009–2012	3,000,000	1,453	10	0	Hospital	Adults	4
[90]	Msyamboza *et al* 2012	Malawi		2007–2010	56,400,000	18,946	299	0	National	All	4
[91]	Mtonga *et al* 2013	Malawi	Blantyre	2010–2010	15,000,000	244	5	100	Hospital	All	3
[93]	Mqoqi *et al* 2004	South Africa		1998–1999		60,908	1,999	0	National	All	1
[92]	Newton *et al* 1996	Rwanda	Butare	1991–1993	2,100,000	455	39	0	Regional	All	4
[94]	Ntakiyiruta 2009	Rwanda		2006–2007	8,000,000		35	57	Hospital	All	1
[95]	Nwafor and Nwafor 2018	Nigeria	Akwa Ibom	2007–2015	36,391,420	1,186	45	100	Hospital	All	3
[96]	Obayo *et al* 2017	Uganda	5 regional hospitals	2002–2011	292,000,000		270	100	Hospital	All	1
[98]	Okobia *et al* 1999	Nigeria	Benin City	1989–1998	104,500,000	816	44	36	Hospital	all	4
[97]	Okongo *et al* 2018	Uganda	Acholi	2013–2016	18,000,000	1,627	17	0	Regional	All	4
[98]	Olson *et al* 2020	Tanzania	Lake Zone	2008–2016	15,000,000	2,772	16	0	Hospital	All	1
[99]	Ouedraogo *et al* 2018	Burkina Faso	North-east	2013–2017	22,000,000	352	61	0	Hospital	All	4
[16]	Parkin *et al* 2021	Zimbabwe	Bulawayo	2011–2015	3,257,000	4,105	321	100	Regional	All	1
[101]	Saeed *et al* 2014	Sudan	Khartoum	2009–2010	8,900,000	6,771	208	0	Regional	All	4
[105]	Somdyla *et al* 2010	South Africa	Eastern Cape	1998–2002	33,000,000	2,501	33	0	Regional	All	1
[102]	Tahtabasi et al 2020	Somalia	Mogadishu	2017–2019	6,300,000	1,306	13	100	Hospital	All	3
[107]	Wabinga *et al* 2009	Uganda	Kyadondo	1995–1997	3,000,000	1,290	37	0	Regional	All	1
[108]	Wabinga *et al* 2001	Uganda	Mbarara	1995–1999	1,460,000	585	50	0	Hospital	All	3
[14]	Wabinga *et al* 2011	Uganda	Kampala	1993–1997	4,833,000	2,523	104	0	Regional	All	1
[110]	Wabinga *et al* 2014	Uganda	Kyadondo	1991–2010	31,529,670	22,494	494	0	Regional	All	1
[105]	Walker *et al* 1999	South Africa	Durban	1995	2,100,000	3,823	109	0	Hospital	All	4
[106]	Warsame *et al* 2021	Somalia	Mogadishu	2019	2,180,000	126	2	0	Hospital	Adults	4
[107]	Wiredu *et al* 2006	Ghana	Accra	1991–2000	14,000,000	3,659	186	0	Hospital	All	4

! = stratified by age and sex

**Table 2.** Treatment data reported in available studies on GC from SSA.

Study reference number	Author	Country	Region/City	Study period	Regional population	Study cancer population	GC cases	Treatment		
								Chemotherapy	Surgery	Radiation
[44]	Afuwape *et al* 2012	Nigeria		2004–2009	25,000,000		49		Curative = 10 Palliative = 13	
[45]	Ahmed *et al* 2011	Nigeria	Zaria	1995–2009	9,375,000		179	57	Total = 155 (D1 = 37 and D2 = 50) Curative = 38 Palliative = 68	
[49]	Asombang *et al* 2014	Zambia	Lusaka	2010–2012	4,384,000		51	6	Palliative = 12	6
[54]	Bang *et al* 2020	Cameroon		2013–2018	19,000,000	1,105	105	Curative = 30 Palliative = 77	Curative =32 Palliative = 16	
[58]	Benamro 2017	South Africa	Durban	2009–2014	15,000,000		131	Curative = 21 Palliative = 40	Curative = 36 Curative = 1 Palliative = 12	17
[73]	Gaskill *et al* 2021	Ghana		2014–2015	30,000,000	2,562	98			
[77]	Ibingira 2001	Uganda	Kampala	1995	964,000		32		Curative = 2 Palliative = 15 Diagnostic/Biopsy = 18	
[6]	Mabula *et al* 2012	Tanzania	Mabula	2006–2011	13,000,000	5,134	232	Curative = 53	Curative = 53 (Total =1 and Partial =52) Palliative =120 Biopsy = 50	
[85]	Martin *et al* 2018	Rwanda		2012–2016	51,000,000		229	Curative = 15 Palliative = 13	Curative = 30 Palliative = 87	
[94]	Ntakiyiruta 2009	Rwanda		2006–2007	8,000,000		35		Curative = 1 Palliative = 34	
[98]	Okobia 1999	Nigeria	Benin City	1989–1998	104,500,000	816	44			

**Table 3.** Mortality data reported in available studies on GC from SSA.

Reference number	Author	Country	Region/City	Study period	GC cases	Outcomes		
						Post-operative complications	Mortality rate (%)	Survival
[42]	Adadey 2020	Ghana	Volta	2012–2014	8		90 All ages = 0.09 60–69 = 1.21 >70 = 0.96	
[45]	Ahmed 2011	Nigeria	Zaria	1995–2009	179	47% (post resection: 1 during index hospitalization)		Median 13.6 months
[49]	Asombang *et al* 2014	Zambia	Lusaka	2010–2012	51			Median 4.6 months
[52]	Bah *et al* 2011	Gambia		1993–1997	52			4.5% 1 year: 17.8% 3 years: 17.8% 5 years: 4.5%
[54]	Bang 2020	Cameroon		2013–2018	105		Mean =5.91 months 85 dead	5 years: 19.0%
[64]	Chokunonga *et al* 2011	Zimbabwe	Harare	1993–1997	144		82 1 year: 37.5% 3-year: 19.0% 5-year: 18.1%	
[74]	Gondos *et al* 2005	Uganda	Kampala	1993–1997	91			0%
[6]	Mabula *et al* 2012	Tanzania	Mabula	2006–2011	232	86	82	5 years survival rate: 6.9% 76 patients followed up 156 lost to follow up
[85]	Martin *et al* 2018	Rwanda		2012–2016	229	31		
[98]	Okobia 1999	Nigeria	Benin City	1989–1998	44		50 (*N* = 26) 7 died within 30 days of hospitalisation	
[109]	Wabinga 2011	Uganda	Kampala	1993–1997	104		100 1 year: 39.1 3 years: 7.5 5 years: 0	Median survival 4.2 months
[107]	Wiredu *et al* 2006	Ghana	Accra	1991–2000	186		100	

## Appendix: GC SR

### Methods – search strategy

Articles applicable to this GC SR were identified by a PubMed, Google Scholar and Web of Science search between 1st January 1995 and 1st June 2022. GC related search terms comprising ‘GC’, ‘stomach neoplasm’, ‘gastric neoplasm’, ‘cancer of the stomach’, ‘GC’ and ‘stomach cancer’ were applied to the search. The SSA country name and the SSA regional was applied with no language restriction.

The final search terms used was ‘*(Cancer OR mass OR malignancy OR adenocarcinoma OR carcinoma OR neoplasm OR tumour) AND (Gastric OR stomach) AND (Botswana OR Burkina Faso OR Burundi OR Cabo Verde OR Cameroon OR central African republic OR Chad OR Comoros OR Democratic Republic of the Congo OR Congo OR Cote D’Ivoire OR Djibouti OR Equatorial Guinea OR Eritrea OR Swaziland OR Ethiopia OR Gabon OR Gambia OR Ghana OR Guinea OR Guinea Bissau OR Kenya OR Lesotho OR Liberia OR Madagascar OR Malawi OR Mali OR Mauritania OR Mauritius OR Mozambique OR Namibia OR Niger OR Nigeria OR Rwanda OR Sao Tome OR Senegal OR Seychelles OR Sierra Leone OR Somalia OR South Africa OR Sudan OR Tanzania OR Togo OR Uganda OR Zambia OR Zimbabwe OR SSA OR Africa)’*

The participants included will be from primary GC studies from 1st January1995 to 1st June 2022 comprising of adult patients above the age of 18 years in SSA who have or had GC. Randomised control trials, cohort studies, case-control studies, and cross-sectional primary data studies of adult GC patients in SSA were included in this analysis. The primary exposure is GC. The primary outcomes of interest are incidence, treatment, and mortality. Studies that describe treatment interventions for GC including both surgery and chemoradiation were included. GC studies conducted outside of SSA or inclusive of non-African countries, paediatric cancer studies, benign gastric tumours, and gastric lymphoma were excluded. Letters to the editor, case reports, case series, narrative reviews, commentaries, perspectives, literature reviews, meta-analyses, medical reports, and non-peer reviewed publications were excluded from the analysis.

All references identified after deployment of the search strategy were exported using Zotero software. All records obtained from the various databases were combined in a single Zotero folder and all duplicates removed. The final search results underwent a title review to assess if the abstracts may contain relevant information for the study. After elimination of non-relevant titles, an abstract review was done prior to a full text review of pertinent studies. A data extraction form was employed on Microsoft Excel to record information on; the surname of primary author, year of publication, country of study, diagnostic method, registry type, study design, age group (age rages including >18 years old), sample size, GC case numbers, overall cancer case number for study population, overall population size, age or gender stratification, treatment type and numbers, and the case fatality rate of GC in the study. References of all relevant articles for additional data sources missed during our search was scanned and included where full texts were retrieved. References of pertinent reviews were also scanned.

5,558 records were identified with the online database search. An initial record screening and removal of duplicated records resulted in 667 studies remaining. Five hundred and thirty-five records were excluded after title screening and 132 study abstracts were screened. After the abstract screening 14 studies were excluded and 123 studies were assessed for eligibility by full text reading and reference reviewal. A total of 85 studies were included in this SR after 38 full text articles were excluded due to outcomes of interest not being reported.

Two reviewers (CG and PM) independently evaluated the titles of studies obtained from the searches. All three reviewers (CG, PM and AR) reviewed abstracts of papers obtained, after which the full texts of potentially eligible papers were retrieved. The three reviewers independently reviewed the full text of each potentially eligible study, compared their results, and resolved any discrepancies by discussion. For duplicate studies published in more than one report, the study reporting the largest sample size was considered. Studies with inaccessible full text either online or from the corresponding author was excluded. Methodological quality and risk of bias of included studies were assessed using the STROBE checklist. https://www.strobe-statement.org/

A SR with descriptive analysis was performed after the final data collection. To determine the incidence of GC, the crude GC case numbers (numerators), the regional population and the population of people with diagnosis of cancer (denominators) were considered. Overall mortality and treatment strategies are reported in crude numbers and percentages. Incidence patterns of GC across the various nations in SSA are summarised after the recalculation of incidence was done.

A narrative summary of findings regarding overall mortality and treatment strategies are reported due to the heterogeneity and paucity of data. All data analyses were conducted using StataSE (version 15.1, College Station, Texas, USA).
